# The Canadian Society of Nephrology methods in developing and adapting clinical practice guidelines: a review

**DOI:** 10.1186/2054-3581-1-5

**Published:** 2014-05-12

**Authors:** Reem A Mustafa, Adeera Levin, Ayub Akbari, Bethany J Foster, Deborah Zimmerman, Gihad E Nesrallah, Greg A Knoll, Jean-Philippe Rioux, Jim Barton, Marcel Ruzicka, Norman Muirhead, Louise Moist, Neesh Pannu, Phil McFarlane, Scott Klarenbach, Susan Samuel, William F Clark, Brenda R Hemmelgarn

**Affiliations:** Department of Clinical Epidemiology and Biostatistics, McMaster University, CanadaHSC Room 2C15 1280 Main Street West, Hamilton, Ontario ON L8S 4 K1 Canada; Department of Internal Medicine, University of Missouri, Kansas City, USA; Department of Medicine, University of British Columbia, Vancouver, British Columbia Canada; Department of Medicine, Division of Nephrology, University of Ottawa, Ottawa, Ontario Canada; Departments of Pediatrics and Epidemiology, Biostatistics, and Occupational Health, McGill University, Montreal, Quebec Canada; Li Ka Shing Knowledge Institute, Keenan Research Centre, St. Michael’s Hospital, and Humber River Hospital, Toronto, Ontario Canada; Ottawa Hospital Research Institute, The Ottawa Hospital, Ottawa, Ontario Canada; Department of Medicine, Nephrology Division, Hôpital du Sacré-Cœur de Montréal, University of Montreal, Montreal, Quebec Canada; Department of Medicine, University of Saskatchewan, Saskatoon, Saskatchewan Canada; Department of Medicine, Western University, London, Ontario Canada; Department of Medicine, Division of Nephrology, University of Alberta, Edmonton, Alberta Canada; Department of Medicine, Li Ka Shing Knowledge Institute, Keenan Research Centre, St. Michael’s Hospital, University of Toronto, Toronto, Ontario Canada; Department of Pediatrics, University of Calgary, Calgary, Alberta Canada; Departments of Medicine and Community Health Sciences, University of Calgary, Calgary, Alberta Canada

**Keywords:** Canadian Society of Nephrology, Clinical practice guideline, Guidelines development, GRADE, Commentary

## Abstract

**Introduction:**

The Canadian Society of Nephrology (CSN) was established to promote the highest quality of care for patients with renal diseases and to encourage research related to the kidney and its disorders. The CSN Clinical Practice Guideline (CPG) Committee develops guidelines with clear recommendations to influence physicians’ practice and improve the health of patients with kidney disease in Canada.

**Review:**

In this review we describe the CSN process in prioritizing CPGs topics. We document the CSN experience using the Grading of Recommendations Assessment, Development and Evaluation (GRADE) approach. We then detail the CSN process in developing de novo CPGs and in adapting existing CPGs and developing accompanying commentaries. We also discuss challenges faced during this process and suggest solutions. Furthermore, we summarize the CSN effort in disseminating and implementing their guidelines. Additionally, we describe recent development and partnerships that allow evaluation of the effect of the CSN guidelines and commentaries.

**Conclusion:**

The CSN follows a comprehensive process in identifying priority areas to be addressed in CPGs. In 2010, the CSN adopted GRADE, which enhanced the rigor and transparency of guideline development. This process focuses on systematically identifying best available evidence and carefully assessing its quality, balancing benefits and harms, considering patients’ and societies’ values and preferences, and when possible considering resource implications. Recent partnership allows wider dissemination and implementation among end users and evaluation of the effects of CPG and commentaries on the health of Canadians.

## Abstract (pt)

**Introduction:**

La mission de la Société canadienne de néphrologie (SCN) est de promouvoir des soins de grande qualité aux patients atteints de maladies rénales et d’encourager la recherche en néphrologie. Le comité des guides de pratique clinique (GPC) de la SCN a pour objectif le développement de lignes directrices comportant des recommandations claires de façon à influencer la pratique médicale et d’améliorer le bien-être des patients atteints de maladies rénales au Canada.

**Revue:**

Dans cette publication, nous décrivons l’approche utilisée par la SCN dans la priorisation des différents sujets abordés dans les GPC. Nous abordons l’expérience de la SCN avec l’utilisation de l’approche GRADE (*Grading of Recommendations Assessment, Development, and Evaluation)*. Nous détaillons également le processus utilisé par la SCN dans le développement de nouveaux GPC et dans la modification des GPC existants. Nous discutons des difficultés rencontrées lors de ce processus tout en suggérant des solutions. Nous résumons les efforts encourus par la SCN pour la diffusion et l’application des lignes directrices. Finalement, nous décrivons les récentes collaborations de la SCN permettant l’évaluation de l’impact des lignes directrices de la SCN.

**Conclusion:**

La SCN suit un processus rigoureux dans l’identification des priorités à aborder dans les GPC. Depuis 2010, la SCN a décidé d’adopter l’approche GRADE pour faire preuve de plus de rigueur et de transparence dans l’élaboration de lignes directrices. Cette approche consiste à identifier toutes les évidences en étudiant leur qualité, en mesurant les risques et les bénéfices, en considérant les valeurs et les préférences pour les patients ainsi que la société et en tenant compte des ressources disponibles. Le partenariat permet une plus grande diffusion des guides de pratique et une évaluation des effets des GPC sur la santé des canadiens.

## Introduction

The Canadian Society of Nephrology (CSN) mission includes promoting the highest quality of care for patients with renal diseases and encouraging research related to the kidney and its disorders. The Clinical Practice Guidelines (CPG) Committee is one of five subcommittees of the CSN, with a mandate to develop CPG with clear recommendations for CSN members [1]. Broadly speaking, CPGs are systematically developed statements to assist practitioners and patients in reaching optimal health care decisions. Their purpose is to “make explicit recommendations with a definite intent to influence clinical practice” [2]. Properly developed CPGs assimilate and translate the abundance of evidence published on a daily basis into recommendations. In doing so, CPGs aim to reduce the use of unnecessary or harmful interventions, and facilitate the treatment of patients to achieve maximum benefit and minimum harm at an acceptable cost. CPGs are not meant to replace, but rather augment, sound medical decision making which takes into account critical elements relevant to patient care including patients’ values and preferences and clinician experience.

The primary objective of the CSN CPG Committee is to develop de novo guidelines for CSN members to improve the health of patients with kidney disease in Canada. High quality CPG development requires considerable resources. While the CSN continues to develop de novo guidelines, collaboration with, and adaptation of, guidelines produced by other relevant organizations including Kidney Disease Improving Global Outcomes (KDIGO), has also been identified as a priority by the CSN membership. To this end the CPG committee has developed principles and a process for the two main activities of the Committee: 1) the development of de novo guidelines, and 2) the adaptation of existing guidelines through a review and written commentary.

The CPG Committee is guided by Terms of Reference that outline roles and responsibilities of the Chair and Members. The Chair is appointed by the CSN President for a 4-year term, and also serves as a member of the CSN Council. Consideration for the CPG committee composition includes representation from other relevant CSN subcommittees, representatives from pediatrics, transplantation and those with expertise in guideline development. As the provision of renal health care services occurs at the provincial level, and in some instances the regional level, consideration of geographic representation on the CPG Committee as well as workgroups in charge of developing de novo CPG and commentaries is also important. All members are required to disclose potential conflicts of interest, including members involved in the working groups for developing de novo CPGs as well as commentaries on existing guidelines. The conflict of interest disclosures are published along with the manuscripts.

In this review we describe the CSN process of adapting existing CPG and developing accompanying commentaries, and document the CSN process and experience of creating de novo CPG using the Grading of Recommendations Assessment, Development and Evaluation (GRADE) approach [3].

## Review

### Prioritizing topics for guideline development – response from the nephrology community

Surveys of the CSN membership (approximately 460 individuals) were undertaken in 2009 and 2013 to get members’ input on the need for guidelines to be produced by the CSN, and the focus of future guidelines. The survey was sent via email to members using Fuildsurveys, and included a combination of multiple choice and open ended questions. In the 2009 survey, 84% of respondents felt that the CSN should continue to produce guidelines, with the majority (72%) indicating that the CSN should not duplicate guidelines produced by other organizations such as KDIGO. A CPG for the management of intensive hemodialysis was identified as a priority topic area with the 2009 survey, and has since been completed [4]. The 2013 survey also elicited membership priority for future guideline development, with the following three top ranked topics: Management of failing kidney transplant patients transitioning to dialysis; management of central venous catheters in hemodialysis, and monitoring and maintenance of arterio-venous access patency. Based on the results of this survey, and consensus from the CPG Committee, the next CSN CPG topic to be produced will be “Management of failing kidney transplant patients transitioning to dialysis”. Although there are limitations to the use of these survey methods to elicit priorities for guideline topics, we did obtain a high response rate and obtained input from across Canada. The CSN CPG Committee will continue to obtain input from the Canadian nephrology community in the future to prioritize guideline development.

### Factors considered when developing or commenting on recommendations

In general the CSN CPGs and Commentaries focus on the effect of an intervention (treatment, performing tests or management strategy) on clinical outcomes that are important to patients (e.g. survival, quality of life, major bleeding) and not on surrogate outcomes (e.g. change in laboratory value or radiologic finding). The CSN CPG developers and Commentary writers are cautious not to draw strong conclusions if an intervention has not been shown to influence important patient outcomes. Since the CSN has adopted the GRADE process its CPGs and Commentaries have been guided by a systematic and transparent “Evidence-to-Recommendations” framework; that includes the following factors: 1) emphasis on available evidence and its quality; 2) balance of benefits and harms; 3) patients’ and societies’ values and preferences and 4) resource implications and feasibility considerations. The quality of the body of evidence is an important consideration as it refers to the confidence guideline developers can place in the available evidence. In other words, the guideline panel confidence that an additional body of evidence addressing the same question is unlikely to change what we already know. Patients’ and societies’ values and preferences are another critical factor that is considered in CSN CPGs and commentaries, with a focus on the specific values in the Canadian context. Where possible resource, feasibility, and cost considerations (from a Canadian perspective), are included, such as the use of Canadian micro-costing data to show resource implications of a recommendation in the Canadian healthcare setting (CSN timing the initiation of chronic dialysis CPG [5]).

### The CSN experience developing clinical practice guidelines

The CSN has developed multiple CPGs since it was established. The CSN process to develop CPGs since adopting GRADE include the following steps:

- Priority setting to determine CPG topics.
- Choose CPG chair/s and establish the working group with representatives from different specialties (e.g. adult and pediatric nephrologists, primary care providers and family physicians, endocrinologists etc.), allied health (e.g. pharmacists, nurses, dietitians etc.), different practice settings (e.g. academic, community, rural etc.) and different geographical and provincial areas. Methodological and guideline development expertise are also considerations when composing CPG working groups.
- Establish CPG development processes and means of communications with clear deadlines for each step.
- Identify target audience and specific topics selection including a focus on treatment vs. diagnosis or a specific population
- Involve end users and stakeholders. This has become a focus for the CSN to ensure CPGs meet the needs of users by developing productive and effective partnerships.
- Generate and select specific answerable questions using PICO format (Patient, intervention, comparison and Outcomes) when appropriate.
- Select outcomes and intervention that are important to patients.
- Conduct a systematic search for relevant evidence including registries of trials and contacting authors of unpublished results that may inform the guidelines.
- Summarize the body of evidence and generate additional information. For example, in the intensive hemodialysis CPG, we surveyed programs to systematically collect information about their experience with the use of “closed connector” devices in patients using a central venous catheter as their access due to paucity of published data. Summarizing the evidence is done using GRADE summary tables (GRADE Evidence Profiles) that can be produced using the Guideline Development Tool [6] (http://www.guidelinedevelopment.org) (formerly called GRADEpro).
- Critically appraise the evidence and its quality, strength or certainty.
- Develop recommendations and determine their strengths. Developing recommendations involves the use of a structured framework that ensures a transparent and systematic process. In GRADE there are two strengths of recommendations; strong or weak/conditional. Each recommendation can be for or against the intervention under question and can be a strong or weak/conditional recommendation. Determining the strength of the recommendations refers to judgments about how confident a guideline panel is that the implementation of a recommendation exerts more desirable than undesirable consequences. Table 1 summarises the implications of the different strengths of recommendations to different end users.
- Finalize wording of recommendations and the remarks/explanations that are required to clarify issues for end-users.
- Peer review. Similar to the CSN commentaries, external peer review with up to three experts in the field is conducted, with revisions as required.
- Disseminating, implementing and evaluating the effects of the CPG. Details about this are described in a later section in this review.
- Update - a clear plan for updating is outlined in each CPG.

**Table 1:** **Implications to strong and weak/conditional recommendations to different end-users**

End-Users	Strong recommendations	Weak/Conditional recommendations
Patients	Most people in this situation would want the recommended course of action and only a small proportion would not	The majority of people in this situation would want the recommended course of action, but many would not
Clinicians	Most patients should receive the recommended course of action	Be more prepared to help patients to make a decision that is consistent with their own values/decision aids and shared decision making
Policy makers	The recommendation can be adapted as a policy in most situations	There is a need for substantial debate and involvement of stakeholders

CSN adheres to international standards in guideline development methods including the AGREE tool and the Institute of Medicine trustworthy guidelines standards [7, 8]. CSN methods are also in line with a recent publication by leaders in the GRADE working group summarizing a comprehensive checklist for a successful guideline enterprise [9].

### The CSN process for adapting existing clinical practice guidelines

Multiple international organizations develop nephrology guidelines. For example, KDIGO is a global guideline organization, with a goal to develop guidelines in every major area of kidney disease [10], from an international perspective. Since its inception in 2003, KDIGO has completed nine comprehensive guidelines. Given the time and resources required in rigorous guideline development, the CSN CPG has focused on collaboration with and adaptation of existing international guidelines like KDIGO, to avoid duplication in effort and to consolidate guideline development. An overview of the Commentaries published and under development is provided in Table 2. To maintain methodological rigor, the CSN CPG subcommittee has developed standards for adapting existing guidelines. Reviews and written commentaries of existing CPG include the following steps:

**Table 2:** **Canadian society of nephrology commentaries on kdigo clinical practice guidelines**

Date (Ref)	Commentary	Status
AJKD 2010 [11]	Canadian Society of Transplantation and Canadian Society of Nephrology on the 2009 KDIGO Clinical Practice Guideline for the Care of Kidney Transplant Recipients	Published
AJKD 2010 [12]	Canadian Society of Nephrology Commentary on the 2009 KDIGO Clinical Practice Guideline for the Diagnosis, Evaluation and Treatment of CKD-Mineral and Bone Disorder (CKD-MBD)	Published
AJKD 2013 [13]	Canadian Society of Nephrology Commentary on the 2012 KDIGO Clinical Practice Guideline for Acute Kidney Injury	Published
AJKD 2013 [14]	Canadian Society of Nephrology Commentary on the 2012 KDIGO Clinical Practice Guideline for Anemia in CKD	Published
AJKD 2014 [15]	Canadian Society of Nephrology Commentary on the 2012 KDIGO Clinical Practice Guideline for Management of BP in Chronic Kidney Disease	In press
AJKD 2014 [16]	Canadian Society of Nephrology Commentary on the 2012 KDIGO Clinical Practice Guidelines for Glomerulonephritis: Management of Glomerulonephritis in Adults	In press
AJKD 2014 [17]	Canadian Society of Nephrology Commentary on the 2012 KDIGO Clinical Practice Guidelines for Glomerulonephritis: Management of Nephrotic Syndrome in Children	In press

- A working group is established consisting of a Chair and co-Chair, appointed by the CSN CPG Committee. A multidisciplinary working group is then formed with invitation to the general CSN membership, as well as invitations to members with known expertise and experience in the topic. The composition varies depending on the guideline, but in general includes nephrologists (adult and pediatric) with clinical and content expertise, relevant physician specialty groups (such as endocrinology, primary care etc.) and members of the allied health community (pharmacists, nursing, dieticians etc.), with consideration for geographical representation across Canada.
- The working group identifies recommendations that require further exposition. Examples of the recommendation that are relevant for interpretation and application may include “challenging” the evidence on which recommendations are based, consideration of resource constraints (frequently excluded from guideline development), and consideration of factors specific to funding and delivery of health care in Canada. A discussion of the “Implications for Canadian Health Care” is created for each identified recommendation. Additionally, recommendations that can be the basis for targeted knowledge translation activities relevant to the local, regional and national context are highlighted.
- Existing guidelines are assessed using the Appraisal of Guidelines Research and Evaluation (AGREE II) [7] instrument as a framework for assessing their quality. Where applicable, guidelines produced by KDIGO may also undergo the application of the ADAPTE process [18], which further considers cultural and organizational contexts. Adaptation of international guidelines considers key questions including variation in: need (prevalence, baseline risk or pre-test probability of health status); directness of the evidence to the local setting, availability of resources and variability in costs; and relative values that patients and societies place on the main benefits and downsides that might lead to different decisions.
- The CSN CPG arranges peer review of the commentary with up to three experts in the field, following which the working group undertakes revisions based on reviewer comments.
- Dissemination and implementation of the Commentaries are as outlined in a later section, including publication in a nephrology journal.

### Developing recommendations and applying grade when evidence quality (confidence in the effect estimates) is low

Some may argue that in certain situations CPGs should not be developed either due to the lack of high quality evidence, or even when high quality evidence exists, when there is close balance between benefits and harms and lack of clear evidence about patients’ values in relations to these benefits or harms. Although guidance is typically more needed when evidence is of low quality, some are concerned that developing recommendations may be misconceived as indorsing this low quality evidence. Hence, outlining the quality of evidence when it is poor is crucial to prevent the promotion of false confidence regarding the best management strategies.

It is generally well recognized that nephrology lags behind other medical sub-specialties in the availability of clinical trials to inform decision making, and hence guidelines. The use of the GRADE process is particularly beneficial in this regard, given the transparency of the process and summary of available evidence. It was clear that reviewers of the CSN CPGs appreciated this transparency and found particular value in the “Evidence-to-Recommendation” tables. In addition to summarizing the evidence, these evidence summaries highlight the shortcomings of the evidence, emphasize the knowledge gaps and identify priorities for future research. There is an emphasis not only on identifying knowledge gaps of the effects of specific interventions but also regarding unknown patients’ values and preferences, physicians’ perceptions, and feasibility and resources consideration. Additionally, comprehensive evidence review and consensus regarding the recommendations promoted valuable new research collaborations to conceive new studies, and strengthened the position of different groups in applying for funding opportunities to support these new studies. An example of an evidence-to-recommendations table is published as a supplementary material of the CSN intensive hemodialysis and timing the initiation of dialysis guidelines [4, 5].

### Challenges faced and suggested solutions

There remain challenges in applying a transparent and rigorous process such as GRADE. These challenges include need for: 1) resources, and in particular those needed to conduct well done systematic reviews, 2) methodological expertise to synthesize the body of evidence and summarize it including experience with appraising and summarizing evidence across different study design, 3) experience in the process used to develop the CPGs such as GRADE, 4) training of the guideline panels to explain the process, 5) communication of the process between clinical and methodological experts to identify and critically appraise the body of the evidence.

The CSN has realized the importance of addressing these challenges by: 1) providing funds to facilitate conducting comprehensive high quality systematic reviews. A process that proved beneficial for the development of CPG despite the limited amount of this funding. The by-product of this process is the opportunity for the guideline panels to coauthor high impact publications and publish in international journals, which offers an additional professional and intellectual incentive and facilitates academic advancement, 2) encouraging the guideline groups to focus the scope of the CPG and maintain the rigor rather than attempting to address a wide range of clinical questions without the availability of the needed resources including panel members time, 3) providing GRADE training to the CSN CPG committee members, panels of different CPG, and members of the CSN utilizing a variety of options including online webinars and in person workshops, 4) ensuring that each guideline working group has clinical and methodological experts, 5) including less experienced panel members in each working group to guide them into the process, 6) facilitating good communication among panel members along the process of developing CPGs including supporting conference calls and in person meetings at national and international nephrology conferences including the CSN annual meeting, 7) reviewing the process after the guideline has been published to identify areas that can be modified in future guideline development. The CSN remains open to incorporate new methodological advances in the area of CPGs development and to help its members keep up to date.

### Dissemination, implementation and evaluation of the effect of CSN guidelines and commentaries

An active implementation process is required to ensure uptake of guidelines and commentaries. Dissemination of the CSN CPG and Commentaries is done through a variety of approaches including publication in peer-reviewed nephrology journals, posting of the full documents on the CSN website with relevant links, posting of summaries and links to other nephrology guideline sites and presentation of the CPG and commentaries in appropriate local, national and international forums. The CSN CPG also partners with the Canadian Kidney Knowledge Translation and Generation Network (CANN-NET) [19], a national organization aimed to improve care for patients with and at risk for chronic kidney disease. CANN-NET plays an active role in the dissemination and implementation of CSN CPG and Commentaries through their links with relevant knowledge users and targeted activities to improve knowledge uptake and dissemination.

The CSN CPGs are viewed favorably internationally due to the rigor, transparency and focused scope. Recently the Saudi Ministry of Health adapted the CSN CPG on timing the initiation of chronic dialysis. The evidence tables summarizing available evidence and discussing its strength simplified the adaptation process. Additionally, the “evidence-to-recommendations” framework facilitated incorporation of local values, preferences and resources that are unique to the Saudi context. We anticipate that use of GRADE will allow other international groups to easily adopt or adapt the CSN recommendations and further facilitates their dissemination.

In addition to dissemination and implementation, evaluation of the impact of the guidelines and commentaries on physician practice and patient outcomes is also a CSN priority. In the past, a formal evaluation of the effect of the guidelines and commentaries on practice change has not been undertaken. However through CANN-NET, and with the availability of data from the Canadian Organ Replacement Registry (CORR), the effectiveness of these knowledge translation activities on process of care and clinical outcomes can be tracked. Canada is fortunate to having universal healthcare with fairly robust health services data at provincial and national level.

## Conclusion

The CSN follows a comprehensive process in developing de novo CPGs and writing Commentaries. In 2010, the CSN adopted GRADE, which enhanced the rigor and transparency of guideline development. This process focuses on systematically identifying all available evidence and carefully assessing its quality, balancing all important patient outcomes including benefits and harms, considering patients’ and societies’ values and preferences, and when possible considering feasibility and resource implications. The CSN also follows a comprehensive and transparent process in identifying priority areas to be addressed in CPGs. This process includes surveying its members and its collaborators’ members to address questions that are of relevance to the national nephrology community. Some of these questions have been identified as priority questions in other countries and led to adaptation of the CSN CPG internationally. One very interesting development is the creation of new partnerships allowing wider dissemination and implementation among end users. Moreover, these partnerships permit evaluating the effects of different knowledge translation products like CPG and commentaries on the health of Canadians. Following this process clearly addresses the CSN mission of promoting the highest quality of care for patients with kidney disease and encouraging research related to the kidney and its disorders.

## Abbreviations

CSN: Canadian Society of Nephrology
CPG: Clinical practice guideline
KDIGO: Kidney disease improving global outcomes
The GRADE process: The grading of recommendations assessment, development and evaluation process
CORR: Canadian Organ Replacement Registry
CANN-NET: Canadian kidney knowledge translation and generation network.
